# Recurring low statistical robustness in orthopaedic surgery: A systematic review of 84 fragility index studies

**DOI:** 10.1002/jeo2.70504

**Published:** 2025-12-01

**Authors:** Hassaan Abdel Khalik, Helena Son, Krishnateja Narayana, Moin Khan, Olufemi Rolland Ayeni

**Affiliations:** ^1^ Division of Orthopaedic Surgery, Department of Surgery McMaster University Hamilton ON Canada; ^2^ Michael G. DeGroote School of Medicine McMaster University Hamilton ON Canada

**Keywords:** evidence‐based medicine, fragility index, orthopaedic surgery, *p* value, research methodology, statistical fragility

## Abstract

**Purpose:**

Increasing attention has been directed towards the fragility index (FI) and reverse fragility index (RFI) in orthopaedic surgery. The purpose of this study was to amalgamate the FI and RFI literature in orthopaedic surgery, and critically appraise its clinical impact.

**Methods:**

Three databases were searched from inception to 22 March 2025, for articles evaluating either the FI or RFI across orthopaedic surgery. Median and mean FI and RFI were presented as ranges with median values. Findings from correlation analyses assessing the impact of study characteristics on FI were consolidated. Citation analysis was performed to assess the uptake of FI/RFI literature.

**Results:**

Eighty‐four studies were included in the final analysis. Sports medicine was the most represented subspeciality (25.0%). Median FI of subspeciality‐specific studies ranged from 1 to 6, and RFI from 3 to 7. Median FI of pathology‐specific studies ranged from 0 to 12, and RFI from 2 to 10. The RFI exceeded the FI in most pathology‐specific studies (93.3%). Decreasing p‐value (88%), increasing sample size (50%) and increasing study power (50%) were commonly found to be associated with increasing fragility index. The median number of citations was 11.5 (interquartile range [IQR], 3.0–27.0) with a median citation density of 3.1 (IQR, 1.2–6.2). Sports medicine publications had the highest collective median citation density of 4.3 (IQR, 1.0–6.6). The h‐index for all included studies was 25, indicating 25 studies had at least 25 citations. Earlier publication year (*p* < 0.001) and increasing journal impact factor (*p* = 0.007) were associated with increased citations.

**Conclusion:**

The majority of fragility index research is concentrated across a few orthopaedic subspecialties with redundant findings indicating low statistical robustness. Recurring methodologic recommendations based on correlation analyses include increasing patient sample size to increase study power. Methodological recommendations from this body of research should be integrated into future original studies to strengthen statistical robustness.

**Level of Evidence:**

Level III.

## Graphical abstract

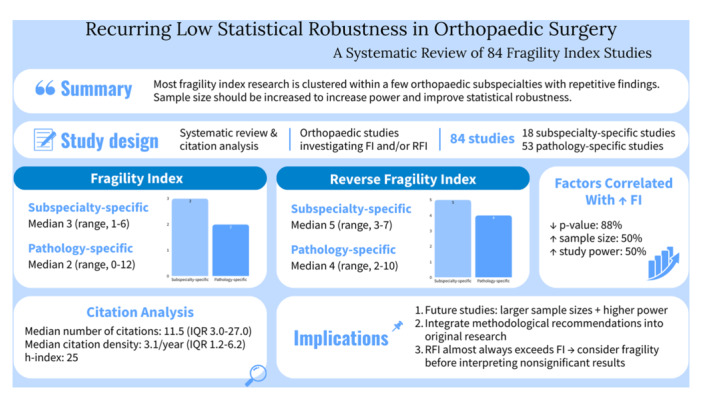

**Abbreviations**
CI: confidence interval
FI: fragility index
IQR: interquartile range
LTFU: lost to follow‐up
NIH: National Institutes of Health
PRISMA: Preferred Reporting Items for Systematic Reviews and Meta‐Analyses
RCT: randomised controlled trial
RFI: reverse fragility index
VIF: variance inflation factor

## INTRODUCTION

Statistical significance has served as a cornerstone of evidence appraisal in clinical research. A value of *p* < 0.05 is often reported as being statistically significant, and thus, conclusions drawn from studies to guide patient care are made from outcomes reporting such a metric. However, reliance on *p*‐values alone may obscure the true robustness of study findings, particularly in randomised control trials (RCTs) and other comparative designs where outcomes are dichotomous. Although established in research, *p*‐values have received criticism for neglecting important factors such as sample size, study power, loss to follow‐up and study design [13, 53, 111].

The fragility index (FI) has emerged as a supplementary metric to address the limitations of the p‐value, quantifying the minimum number of outcome event reversals required to change a statistically significant result to one that is non‐significant [30, 103]. A low FI suggests that an outcome is highly sensitive to small changes in events, raising concern for the statistical stability of study conclusions. The reverse fragility index (RFI) applies the same principle in reverse, indicating the number of events that need to change to make a non‐significant result significant [5, 44]. The application of the FI and RFI shifts the focus from arbitrary thresholds to the actual resilience of statistical outcomes, broadening the lens through which study results can be evaluated [24, 58, 80]. The fragility index has become widely applied across medical disciplines, including gynaecologist, cardiology, vascular surgery and oncology [20, 50, 83, 101]. The use of FI has become particularly relevant in fields characterised by relatively small samples and limited event rates [10, 14, 27, 90]. Consequently, methodological investigations are increasingly incorporating FI and RFI as tools to assess the credibility of evidence and inform clinical decision‐making.

In orthopaedic surgery research, the interest in fragility‐based metrics is growing; however, the literature remains fragmented as to the thresholds for acceptable fragility, interpretation and clinical relevance of FI, as well as the comparability of fragility metrics across studies. Studies have investigated FI within specific orthopaedic subspecialties, such as sports medicine, spine surgery, and arthroplasty, with wide variation in focus, methodology and interpretation [42, 59, 89, 109]. Further, some studies attempt to correlate FI with trial characteristics such as sample size or journal impact factor, while others investigate the relationship between the FI and the number of patients lost to follow‐up (LTFU) – a critical but often underreported variable that may undermine study validity if exceeding the FI itself [2, 27, 43, 73]. Further, there is no consensus on what constitutes an ‘acceptable’ or ‘robust’ FI. This lack of a formal threshold complicates the clinical interpretation and integration of FI values across orthopaedic studies, even within the same subspeciality.

The heterogeneity in study designs and reporting standards makes it difficult to draw generalisable conclusions about the utility and implications of the FI and RFI in orthopaedic research [27, 72, 98, 102]. Without consistent methodologies or comparative analyses across subspecialties, it remains unclear as to whether FIs can meaningfully inform evidence‐based orthopaedic practice. The need for a synthesised and comprehensive appraisal of fragility‐based metrics across orthopaedic subspecialities is pressing, both to guide future orthopaedic research and to assist clinicians in interpreting the literature with appropriate caution.

The aim of this systematic review is threefold. First, it aimed to assess and compare the fragility index and reverse fragility index across a wide range of orthopaedic surgery subspecialities. Second, it intended to identify factors that are routinely identified as being associated with an increased FI, such as sample size, event rates, or other methodological recommendations. Third, it sought to assess the uptake of the fragility index literature across orthopaedic subspecialties using a citation analysis. It was hypothesised that (1) the FI will be concentrated around a similar value, with the RFI often exceeding the FI, (2) increasing sample size will be the most common significant correlation associated with larger FI values, and (3) sports medicine will be the most active subspeciality publishing on the subject matter.

## METHODS

This review was performed according to the guidelines set out by the Cochrane Handbook and is reported according to the Preferred Reporting Items for Systematic Reviews and Meta‐analyses (PRISMA) [38, 70].

Three electronic databases, MEDLINE, EMBASE and the Cochrane Database of Systematic Reviews, were searched from database inception to 22 March 2025 for literature assessing the fragility index across various orthopaedic subspecialties and pathologies. The search terms included ‘fragility index’, ‘fragility quotient’ and ‘statistical fragility’ (Supporting Information: Table 1).

Inclusion criteria were (1) prior studies with the explicitly stated primary aim of assessing the fragility index and/or the reverse fragility index across various orthopaedic subspecialties or pathologies, (2) any level of evidence and 3) published in the English language. Studies were ineligible for inclusion if (1) the fragility index was assessed as a secondary aim, (2) only the combined FI was reported with no stratification by FI and RFI or (3) the study assessed registry‐based research.

### Study screening

Two authors (HS and KN) independently screened the titles and abstracts of the identified studies using the eligibility criteria. Disagreements were automatically advanced to the full‐text review stage to prevent any premature exclusions. If needed, a senior author (HAK) resolved any full‐text disagreement.

### Data abstraction from included studies

Two reviewers (HS and KN) abstracted data in duplicate from the included studies into an input form in Covidence (Veritas Health Innovation, Melbourne, Australia) which was designed a priori. Conflicts were resolved through consensus, with a senior author (HAK) consulted to resolve remaining discrepancies. The following characteristics were abstracted from each study: (1) journal name and impact factor, (2) country of publication, (3) subspeciality, (4) area of study, (5) whether solely RCTs were evaluated, (6) number of outcomes evaluated, (7) fragility and/or reverse fragility indices as reported by study and (8) findings of correlation analyses.

Studies were categorised as either subspeciality‐specific or pathology‐specific. Subspeciality‐specific studies sought to assess the FI/RFI of an entire orthopaedic subspeciality, while pathology‐specific studies assessed a particular pathology within a subspeciality.

### Outcomes

The primary outcomes of this study were the fragility and reverse fragility indices across the orthopaedic surgery literature, and stratified by subspeciality. Second, correlation analyses conducted in included studies were assessed for factors significantly associated with increased FI (e.g., sample size, event rates, *p*‐value), which may inform future methodological recommendations. Third, a citation analysis was conducted to assess the uptake of fragility index literature in orthopaedic surgery.

### Statistical analysis

#### Fragility and reverse fragility indices

To avoid pooling of heterogeneous outcomes, the fragility and reverse fragility indices across eligible studies were presented as ranges along with the 50th percentile value (median). This was performed separately for studies presenting their FIs/RFIs as means and medians, respectively. Conversion of descriptive statistics to a single measure of central tendency (e.g., median) was not performed to avoid inappropriate assumptions of normality and resultant inaccurate conversions.

#### Correlation analyses of included studies

To identify strategies for designing statistically robust studies, prior publications assessing the FI across a given subject matter often conduct correlation analyses between various study characteristics (e.g., sample size) and their association with the FI. As such, this review amalgamated findings from the correlation analyses conducted by eligible studies, along with the statistical methodology utilised. More specifically, this study tabulated whether or not certain characteristics were significantly correlated with the FI. If a study performed multiple types of correlation analyses, findings from the adjusted model (e.g., multivariable regressions) were included in the final analysis.

#### Citation analysis

Total citation counts of included studies were obtained from Google Scholar on 20 May 2025. The citation density was calculated for all studies, wherein total citations are divided by years published (i.e., total citations divided by years since publication equals the citation density). Studies published in 2025 were divided by one year, and so forth. Normality of data was assessed using the Shapiro–Wilk or Kolmogorov–Smirnov tests for sample sizes containing <50 or ≥50 data points, respectively. Means and standard deviations or medians and interquartile ranges were used as descriptive statistics for normally and non‐normally distributed data, respectively. Similarly, comparison of descriptive statistics was performed using appropriate methods based on distribution of data. The h‐index of included studies was reported along with the h5‐index, which captures publications over the last 5 years (2020 to end of 2024) [39]. The h‐index was calculated by ranking all eligible papers by their citation count and identifying the threshold where the paper′s rank equals its citation count. A multivariable linear regression was conducted with total citations as the dependent variable and (1) publication year, (2) journal impact factor, (3) number of studies included, (4) inclusion of only RCTs, (5) subspeciality‐ or pathology‐specific and (6) total number of outcomes assessed as the independent variables. Six independent variables were selected to ensure at least 10 datapoints were available per predictor variable, preventing model overfitting [36]. Collinearity was assessed using the variance inflation factor (VIF) wherein values greater than 10 indicate severe collinearity [17].

All statistical analyses were performed on SPSS with data visualisation performed on both SPSS and Microsoft Excel. A value of *p* < 0.05 was considered significant.

## RESULTS

### Study characteristics

**Figure 1:**
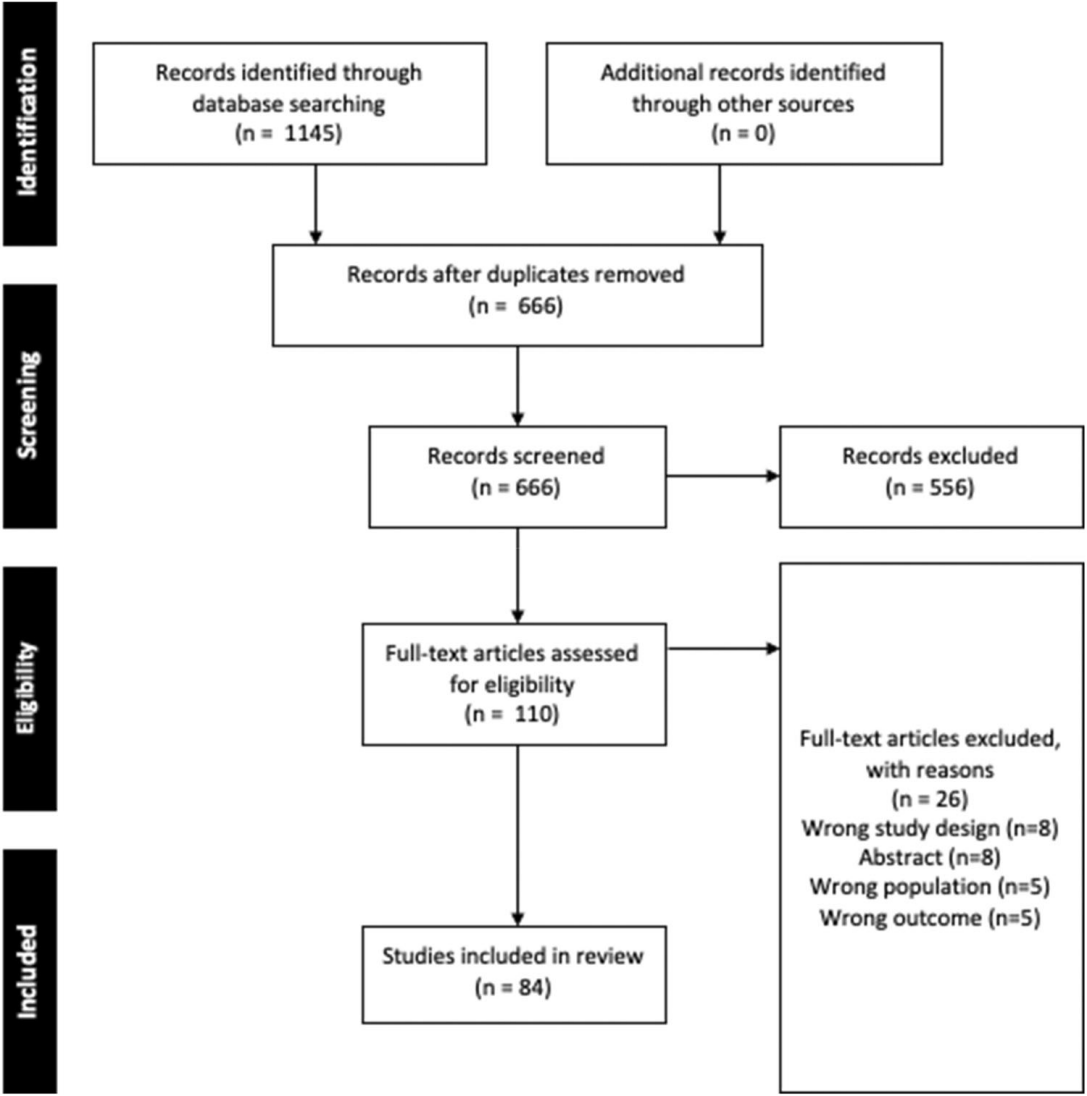
Preferred Reporting Items for Systematic Reviews and Meta‐Analyses.

**Figure 2:**
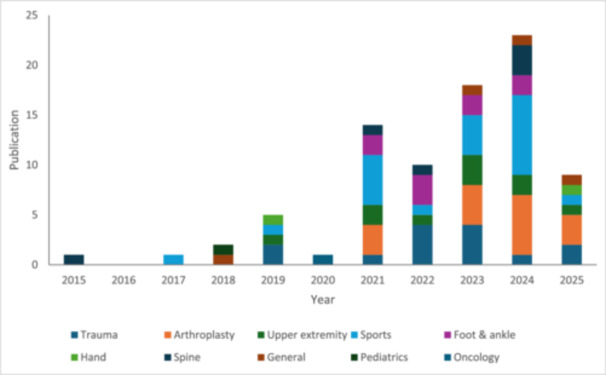
Histogram of publications to 22 March 2025.

The search initially identified 1145 studies, with 84 studies included in the final analysis (Figure 1). The most commonly represented orthopaedic subspeciality was sports medicine (25.0%) followed by arthroplasty (19.0%) and trauma (16.7%) (Table 1). The majority of studies were published from the United States (88.1%). The top three represented journals were the *Journal of Arthroplasty* (9.5%), *Journal of Shoulder and Elbow Surgery* (8.3%) and the *American Journal of Sports Medicine* (8.3%). Most studies solely included RCTs (81%), and the majority of studies were pathology‐specific (77.4%). There continues to be an increase in publications over the last several years (Figure 2). Characteristics of included studies can be found in Table 2.

**Table 1:** Overview of included studies.

	*n* (%)
Subspeciality	
Sports	21 (25.0)
Arthroplasty	16 (19.0)
Trauma	14 (16.7)
Upper extremity	10 (11.9)
Foot & ankle	9 (10.7)
Spine	6 (7.1)
General	4 (4.8)
Hand	2 (2.4)
Paediatrics	1 (1.2)
Oncology	1 (1.2)
Total	84
Country	
United States	74 (88.1)
Canada	4 (4.8)
Ireland	3 (3.6)
China	1 (1.2)
India	1 (1.2)
Italy	1 (1.2)
Journals	
*J Arthroplasty*	8 (9.5)
*J Shoulder Elbow Surg*	7 (8.3)
*Am J Sports Med*	7 (8.3)
*Orthop J Sports Med*	6 (7.1)
Other	37 (66.7)
Level of evidence included	
RCTs only	68 (81)
RCTs & non‐RCTs	16 (19)
Pathology‐ vs. subspeciality‐specific	
Pathology‐specific	65 (77.4)
Subspeciality‐specific	19 (22.6)
Outcomes included	
All	8830
Significant	2348 (26.6)
	6482 (73.4)

Abbreviation: RCTs, randomised controlled trials.

**Table 2:** Included study‐specific characteristics.

Author (year)	Journal name	Country	Subspeciality	Included studies, *n*	Reporting FI, RFI or both?	Significant outcomes, *n*	Non‐significant outcomes, *n*	Total outcomes, *n*
Anaspure et al. (2024) [1]	*Foot Ankle Orthop*	United States	Foot & ankle	11	FI	24	—	24
Bhale et al. (2024) [4]	*Hip Int*	United States	Sports	12	Both	8	17	25
Bragg et al. (2024) [5]	*Am J Sports Med*	United States	Foot & ankle	9	RFI	—	46	46
Brown et al. (2024) [7]	*J Am Acad Orthop Surg*	United States	General	108	Both	45	147	192
Byrne et al. (2024) [8]	*Orthop J Sports Med*	United States	Sports	29	Both	10	38	48
Carroll et al. (2022) [9]	*J Shoulder Elbow Surg*	United States	Trauma	10	FI	10	0	10
Chan et al. (2023) [10]	*Orthop J Sports Med*	United States	Sports	8	Both	51	21	72
Checketts et al. (2018) [11]	*J Bone Joint Surg Am*	United States	General	72	FI	72	—	72
Constant et al. (2022) [15]	*Am J Sports Med*	United States	Sports	22	Both	25	50	75
Cordero et al. (2023) [16]	*J Arthroplasty*	United States	Arthroplasty	23	Both	11	80	91
Davey et al. (2023) [18]	*Am J Sports Med*	Ireland	Sports	21	FI	NR	NR	NR
Doyle et al. (2022) [21]	*J ISAKOS*	Ireland	Foot & ankle	51	Both	11	235	246
Doyle et al. (2023) [22]	*JSES Rev Rep Tech*	Ireland	Arthroplasty	19	Both	11	74	85
Dworsky—Fried et al. (2024) [23]	*Knee Surg Sports Traumatol Arthrosc*	Canada	Sports	11	Both	9	10	19
Ehlers et al. (2021) [24]	*Am J Sports Med*	United States	Sports	18	Both	21	93	114
Ehlers et al. (2021) [25]	*Orthop J Sports Med*	United States	Sports	15	Both	7	41	48
Ekhtiari et al. (2021) [26]	*J Arthroplasty*	Canada	Arthroplasty	34	FI	34	—	34
Evaniew et al. (2015) [27]	*Spine J*	Canada	Spine	40	FI	40	—	40
Fackler et al. (2022) [28]	*Orthop J Sports Med*	United States	Upper extremity	22	Both	16	58	74
Fackler et al. (2022) [29]	*Foot Ankle Int*	United States	Foot & ankle	17	Both	6	34	40
Forrester et al. (2020) [31]	*J Am Acad Orthop Surg Glob Res Rev*	United States	Oncology	23	Both	12	36	48
Forrester et al. (2021) [32]	*J Am Acad Orthop Surg Glob Res Rev*	United States	Trauma	128	Both	110	435	545
Giakas et al. (2025) [33]	*Orthopedics*	United States	Arthroplasty	11	Both	5	58	63
Gonzalez et al. (2024) [34]	*J Arthroplasty*	United States	Arthroplasty	10	RFI	—	10	10
Gupta et al. (2022) [35]	*World Neurosurg*	United States	Spine	6	Both	2	11	13
Herndon et al. (2021) [37]	*Arthroplast Today*	United States	Arthroplasty	104	Both	91	382	473
Hohmann et al. (2024) [40]	*J Arthroplasty*	United States	Arthroplasty		Both	75	328	403
Imbergamo et al. (2023) [41]	*J Shoulder Elbow Surg*	United States	Upper extremity	17	Both	11	25	36
Khan et al. (2017) [42]	*Am J Sports Med*	Canada	Sports	48	FI	48	—	48
Khormaee et al. (2018) [45]	*J Pediatr Orthop*	United States	Paediatrics	17	FI	17	—	17
Koehne et al. (2025) [47]	*Hand (N Y)*	United States	Hand	15	Both	9	37	46
Koehne et al. (2025) [46]	*J Orthop*	United States	Trauma	13	Both	15	21	36
Kyriakides et al. (2022) [48]	*Eur J Trauma Emerg Surg*	United States	Trauma	25	Both	17	31	48
Lawrence et al. (2024) [49]	*Arthroscopy*	United States	Sports	19	Both	5	50	55
Locke et al. (2024) [52]	*J Arthroplasty*	United States	Arthroplasty	19	Both	9	37	46
Locke et al. (2025) [51]	*J Shoulder Elbow Surg*	United States	Upper extremity	38	Both	17	50	67
Maldonado et al. (2021) [54]	*Arthroscopy*	United States	Sports	8	FI	8	—	8
Marasco et al. (2021) [55]	*Knee Surg Sports Traumatol Arthrosc*	Italy	Foot & ankle	4	FI	8	—	8
Mazzucco et al. (2024) [56]	*J Arthroplasty*	United States	Arthroplasty	8	RFI	—	16	16
McCormick et al. (2021) [58]	*J Shoulder Elbow Surg*	United States	Arthroplasty	13	Both	4	35	39
Megafu et al. (2022) [60]	*Injury*	United States	Trauma	34	Both	30	121	151
Megafu et al. (2023) [64]	*J Shoulder Elbow Surg*	United States	Upper extremity	7	Both	6	18	24
Megafu et al. (2023) [62]	*Eur J Orthop Surg Traumatol*	United States	Trauma	9	Both	25	73	98
Megafu et al. (2023) [61]	*Foot (Edinb)*	United States	Foot & ankle	19	Both	30	49	79
Megafu et al. (2024) [63]	*Arthroscopy*	United States	Sports	11	Both	32	186	218
Megafu et al. (2024) [59]	*Arch Orthop Trauma Surg*	United States	Spine	35	Both	130	563	693
Megafu et al. (2025) [65]	*J Back Musculoskelet Rehabil*	United States	Trauma	30	Both	62	188	250
Mian et al. (2023) [66]	*Injury*	United States	Trauma	6	Both	5	40	45
Mian et al. (2024) [67]	*Iowa Orthop J*	United States	Sports	17	Both	26	86	112
Milto et al. (2023) [68]	*J Foot Ankle Surg*	United States	Foot & ankle	18	FI	19	—	19
Minhas et al. (2023) [69]	*Eur J Trauma Emerg Surg*	United States	Trauma	10	Both	12	99	111
Morris et al. (2022) [71]	*World J Orthop*	United States	Trauma	15	FI	91	—	91
Muthu et al. (2021) [72]	*Spine (Phila Pa 1976)*	India	Spine	70	FI	70	—	70
Oeding et al. (2024) [74]	*Am J Sports Med*	United States	Sports	16	Both	14	34	48
Ortiz—Babilonia et al. (2024) [75]	*Spine (Phila Pa 1976)*	United States	Spine	25	Both	5	8	13
Parisien et al. (2019) [77]	*J Am Acad Orthop Surg*	United States	Sports	102	Both	98	241	339
Parisien et al. (2019) [76]	*J Orthop Trauma*	United States	Trauma	198	Both	235	540	775
Parisien et al. (2021) [81]	*Arthrosc Sports Med Rehabil*	United States	Upper extremity	198	Both	74	283	357
Parisien et al. (2021) [82]	*JB JS Open Access*	United States	Sports	52	Both	33	117	150
Parisien et al. (2021) [78]	*J Am Acad Orthop Surg Glob Res Rev*	United States	Foot & ankle	51	Both	32	145	177
Parisien et al. (2021) [79]	*Cartilage*	United States	Sports	19	Both	23	37	60
Parisien et al. (2021) [80]	*Am J Sports Med*	United States	Upper extremity	19	Both	6	45	51
Pearsall et al. (2023) [84]	*J Am Acad Orthop Surg*	United States	General	8	Both	8	42	50
Polisetty et al. (2024) [85]	*J Exp Orthop*	United States	Arthroplasty	21	Both	5	66	71
Poursalehian et al. (2024) [86]	*J Am Acad Orthop Surg*	United States	General	160	Both	39	29	68
Proal et al. (2024) [87]	*Eur Spine J*	United States	Spine	32	Both	96	144	240
Ruelos et al. (2023) [89]	*Knee Surg Sports Traumatol Arthrosc*	United States	Sports	16	RFI	—	20	20
Ruzbarsky et al. (2019) [91]	*J Shoulder Elbow Surg*	United States	Trauma	15	FI	15	—	15
Ruzbarsky et al. (2019) [90]	*J Hand Surg Am*	United States	Hand	5	FI	5	—	5
Ruzbarsky et al. (2019) [92]	*J Shoulder Elbow Surg*	United States	Upper extremity	30	FI	30	—	30
Sequeira et al. (2023) [94]	*Orthop J Sports Med*	United States	Upper extremity	14	Both	7	41	48
Sequeira et al. (2023) [93]	*Arthroplast Today*	United States	Arthroplasty	32	Both	70	233	303
Shi et al. (2023) [95]	*J Bone Joint Surg Am*	United States	Arthroplasty	10	RFI	—	10	10
Singh et al. (2025) [96]	*Orthop J Sports Med*	United States	Sports	6	Both	6	26	32
Sudah et al. (2023) [99]	*Arthroscopy*	United States	Sports	54	RFI	—	54	54
Sudah et al. (2024) [100]	*HSS J*	United States	Upper extremity	6	RFI	—	6	6
Williamson et al. (2024) [104]	*J Clin Med*	United States	Arthroplasty	10	Both	3	35	38
Xu et al. (2022) [105]	*Foot Ankle Orthop*	United States	Foot & ankle	8	Both	1	11	12
Yendluri et al. (2024) [106]	*J Arthroplasty*	United States	Arthroplasty	17	Both	13	45	58
Yendluri et al. (2024) [107]	*J Orthop Trauma*	United States	Trauma	71	Both	47	150	197
Yendluri et al. (2024) [109]	*J Shoulder Elbow Surg*	United States	Upper extremity	18	Both	13	46	59
Yendluri et al. (2024) [108]	*Cartilage*	United States	Sports	21	Both	32	123	155
Zabat et al. (2024) [110]	*J Arthroplasty*	United States	Arthroplasty	7	Both	16	22	38
Zhang et al. (2023) [112]	*Injury*	China	Trauma	10	FI	10	—	10

Abbreviations: FI, fragility index; NR, not reported; RFI, reverse fragility index.

### Fragility and reverse fragility index

**Figure 3:**
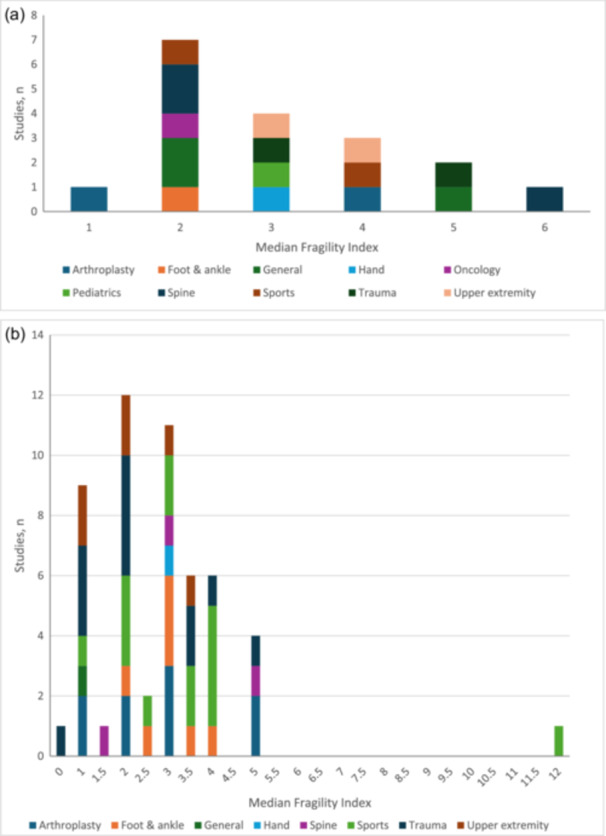
(a) Histogram of median fragility index across subspeciality‐specific studies. (b) Histogram of median fragility index across pathology‐specific studies.

Across 18 subspeciality‐specific studies, the median FI was 3 (range, 1–6), while the median RFI was 5 (range, 3–7) across nine studies (Figure 3a). Nine studies reported both median FI and RFI, with 88.9% reporting an RFI greater than the FI. Similarly, three studies reported both mean FI and RFI, with two of three studies (66.7%) reporting an RFI greater than the FI. Further details regarding median and mean FI and RFI for subspeciality‐specific studies can be found in Table 3.

**Table 3:** Fragility index and reverse fragility index across subspeciality‐specific studies.

	Fragility index					Reverse fragility index			
	*n* eligible	Minimum	50th percentile	Maximum		*n* eligible	Minimum	50th percentile	Maximum
Studies reporting as median									
All	18	1	3	6		**9**	**3**	**5**	**7**
Arthroplasty	2	1	2.5	4		1	—	7^a^	—
Foot and ankle	1	—	2^a^	—		—	—	—	—
General	3	2	2	5		2	3	4	5
Hand	1	—	3^a^	—		—	—	—	—
Oncology	1	—	2^a^	—		1	—	4^a^	—
Paediatric	1	—	3^a^	—		—	—	—	—
Spine	3	2	2	6		1	—	6^a^	—
Sports	2	2	3	4		1	—	6^a^	—
Trauma	2	3	4	5		2	5	5	5
Upper extremity	2	1	3.5	4		1	—	5^a^	—
Studies reporting as mean									
All	**3**	5	7	11		4	3.7	3.3	5.0
Arthroplasty	1	—	5.6^a^	—		1	—	7^a^	—
Oncology	1	—	11^a^	—		—	—	4	—
Sports	—	—	—	—		1	—	3.7^a^	—
Trauma	1	–	5^a^	–		1	–	5.9^a^	–

^a^ Only one study.

Across 53 pathology‐specific studies, the median FI was 2 (range, 0–12), while the median RFI was 4 (range, 2–10) (Figure 3b). Forty‐five studies reported both median FI and RFI, with 93.3% reporting an RFI greater than the FI. Thirteen studies reported both mean FI and RFI, with 76.9% reporting an RFI greater than the FI. Table 4 provides further details regarding median and mean FI and RFI for pathology‐specific.

**Table 4:** Fragility index and reverse fragility index across pathology‐specific studies reported.

	**Fragility index**					**Reverse fragility index**			
	***n* eligible**	**Minimum**	**50**th **percentile**	**Maximum**		***n* eligible**	**Minimum**	**50**th **percentile**	**Maximum**
Studies reporting as median									
All	53	0	2	12		52	2	4	10
Arthroplasty	9	1	3	5		12	3	4.875	7
Foot and ankle	7	2	3	4		6	2.5	4	8
General	1	—	1^a^	—		1	—	4^a^	—
Hand	1	—	3^a^	—		1	—	5^a^	—
Spine	3	1.5	3	5		3	2	5	9
Sports	14	1	3.25	12		14	3	5	9.5
Trauma	12	0	2	5		8	4	4	10
Upper extremity	6	1	2	3.5		7	3	4	7
Studies reporting as mean									
All	15	1.3	3.2	6.9		13	3.0	4.4	9.2
Arthroplasty	4	1.3	5.3	6.0		4	5.5	6.4	9.2
Foot and ankle	1	—	2.3^a^	—		1	—	3	—
Sports	5	1.5	4.6	6.9		4	3.1	3.5	4.4
Trauma	2	2.0	2.3	2.3		1	—	3.95^a^	—
Upper extremity	3	1.3	2.0	3.2		3	3.0	4.8	5.0

^a^ Only one study.

### Summary of correlation analyses

**Figure 4:**
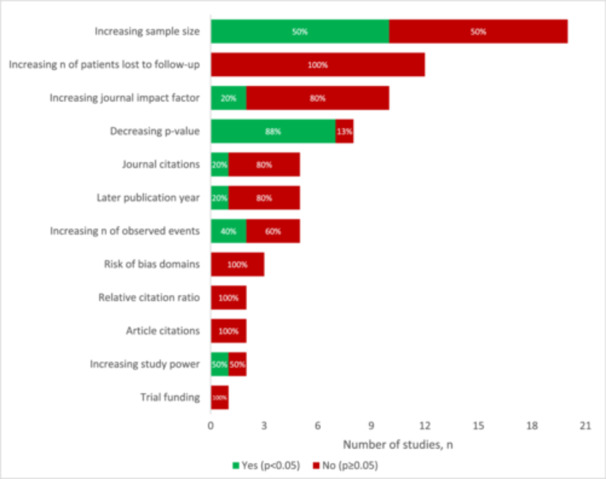
Findings from correlation analyses across included studies. Items significantly associated with the fragility index are illustrated in green (*p* < 0.05), while items not significantly associated with the fragility index are illustrated in red (*p* ≥ 0.05).

Twenty‐one studies (25%) performed correlation analyses across various study/publication characteristics and the fragility index. The Pearson correlation was most common (52%), followed by the Spearman correlation (38%) and multivariable regressions (10%). The most commonly assessed characteristics were increasing sample size (95%), number of patients lost to follow‐up (57%) and journal impact factor (48%) (SDC Table 2). Decreasing p‐value (88%), increasing sample size (50%) and increasing study power (50%) had the highest proportion of significant correlations across the FI (Figure 4).

### Citation analysis

**Figure 5:**
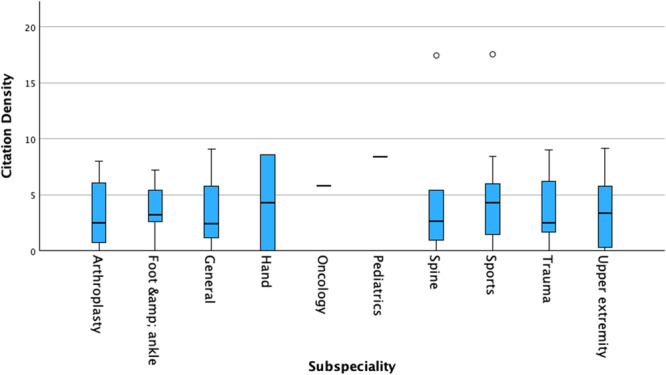
Distribution of citation density by subspeciality.

The median journal impact factor across all studies was 2.7 (interquartile range [IQR], 2.0–3.4). The median number of citations across all studies was 11.5 (IQR, 3.0–27.0) with a median citation density of 3.1 (IQR, 1.2‐6.2) (Table 5). Arthroplasty articles had the highest median journal impact factor of 3.4 (IQR, 2.4–4.2), while sports medicine and foot & ankle articles tied for the highest median citation count of 13. Excluding subspecialties with only one published article, sports medicine presented with the highest median citation density of 4.3 (IQR, 1.0–6.6). Independent‐samples Kruskal–Wallis test demonstrated no significant difference in citation density across subspecialties (*p* = 0.921) (Figure 5). The h‐index for all included studies was 25, indicating there are 25 studies with at least 25 citations. The h5‐index was 22.

**Table 5:** Citation analysis descriptive characteristics.

	Journal impact factor, median (IQR)	Citations, *n* (%)	Citations, median (IQR)	Citation density, median (IQR)
All	2.7 (2.0–3.4)	1656 (100)	11.5 (3.0–27.0)	3.1 (1.2–6.2)
Sports	3.3 (2.4–4.2)	452 (29)	13.0 (1.5–30.0)	4.3 (1.0–6.6)
Arthroplasty	3.4 (2.0–3.4)	178 (11)	5.0 (0.8–23.5)	2.5 (0.4–6.6)
Trauma	2.0 (1.6–2.2)	225 (14)	12.5 (3.3–‐25.5)	2.5 (1.5–6.5)
Upper extremity	2.9 (2.4–2.9)	161 (10)	11.0 (0.8–30.3)	3.4 (0.3–6.5)
Foot and ankle	2.6 (1.8–3.5)	125 (8)	13.0 (8.0–20.5)	3.3 (2.3–6.2)
Spine	2.7 (2.0–3.3)	238 (14)	8.5 (1.5–68.3)	2.6 (0.8–8.4)
General	2.6 (2.6–4.0)	85 (5)	6.0 (1.3–56.5)	2.4 (0.6–7.5)
Hand	2.1^a^	60 (4)	30 (N/A)	4.3 (N/A))
Paediatrics	1.4^a^	67 (4)	67	8.4
Oncology	2^a^	35 (2)	35	5.8

Abbreviations: IQR, interquartile range; N/A, not applicable.

^a^ Only one study.

Multivariable linear regression modelling demonstrated a significant inverse relationship with publication year and total citations (*p* < .001) and a significant direct correlation with journal impact factor and total citations (*p* = 0.007). Number of included studies, sole inclusion of RCTs, focus on pathology‐specific presentations and total number of outcomes assessed were not significantly associated with total citations (Table 6).

**Table 6:** Multivariable linear regression.

Parameter	Beta	95% CI	*p* value
Publication year	−11.740	−14.058 to −9.421	<0.001
Journal impact factor	5.907	1.647–10.167	0.007
Number of included studies	.035	−0.134 to 0.205	0.677
Inclusion of RCTs only RCT and non‐RCT = 0, RCT only = 1	3.338	−6.644 to 13.32	0.507
Pathology‐ vs. subspeciality‐specific pathology = 0, general = 1	3.893	−8.794 to 16.579	0.542
Total number of outcomes	−.003	−0.04 to 0.034	0.867

Abbreviations: CI, confidence interval; RCT, randomised controlled trial.

## DISCUSSION

Despite ongoing research on the fragility index in orthopaedic surgery, its clinical relevance and applicability to future research remains unclear. Heterogeneous study designs coupled with siloed efforts within subspecialties render definitive recommendations and future direction challenging. As such, the purpose of this review was to consolidate the fragility index literature in orthopaedics, highlight key methodologic recommendations as well as critically evaluate the impact of this methodologic area of study. The majority of eligible studies reported their median FI which ranged from 1 to 6 across subspeciality‐specific studies, and from 0 to 12 across pathology‐specific studies. Concerningly, 93% of studies reporting both median FI and RFI found that the latter exceeded the former. Correlation analyses across included studies most consistently demonstrated significant associations between increasing fragility index and decreasing *p*‐value, increasing sample size and increasing study power. This review's regression analysis demonstrated that increasing citations were driven by older publication date as well as being published in higher impact journals.

### Fragility and reverse fragility index

This review demonstrated that the FI and RFI are largely comparable across orthopaedic subspecialties. More importantly, this review highlights that the RFI almost always exceeded the FI. Though there lacks consensus regarding the ‘threshold′ for what is considered a statistically robust FI, a more practical use of the FI and RFI is their comparison to patients lost to follow‐up (LTFU). An oversimplification of the relationship between LTFU and the FI is that if the LTFU exceeds the FI, then the trial is likely to have its significant findings reversed after the inclusion of lost patients. Expectedly, this line of reasoning has been called into question as it assumes (1) *all* patients lost to follow‐up would have the worst case outcome, (2) all would be from the intervention group and (3) the denominator of patients does not change despite adding new participants [2, 73]. As demonstrated by Oeding et al., a hypothetical trial with an FI of four required the addition of *seven* patients to reverse the significance of findings [73]. Another important methodologic consideration surrounds the heterogeneous reporting of FI and RFI. Specifically, both median and mean values are reported in the literature, without consistent assessment of normality. Not only does this render comparison across studies challenging, but this may artificially increase the FI considering it′s generally right‐skewed, as further demonstrated by this study (Figure 3a,b). Ultimately, the standalone use of the FI and RFI remains fraught with methodologic concerns and a lack of consensus regarding what is deemed ‘robust′.

### Correlation analysis

Irrespective of the methodologic limitations of the fragility index, its underlying measure of statistical robustness should still be a leading priority in the design of RCTs. Expectedly, decreasing *p*‐value (88%), increasing sample size (50%), and increasing study power (50%) were all found to be associated with increasing fragility index. Unfortunately, surgical trials are more resource intensive and invasive relative to pharmacologic trials, which renders the aforementioned objectives challenging. Strategies to achieve high‐powered RCTs include multi‐centre collaboration as well as the use of innovative trial designs. Most notably are factorial trials, which have been commonly utilised in pharmacologic trials, and increase a study′s efficiency by assessing the effects of two or more interventions simultaneously [57]. For example, a recent pilot trial by Slobogean et al. utilised this trial design to assess the impact of both surgical intervention (sliding hip screw versus cancellous screw) as well as vitamin D supplementation (vitamin D3 versus placebo) on a composite outcomes of patient complications [97]. Further, multi‐centre trial collaboration remains an untapped resource, with Brophy et al. demonstrating that orthopaedic surgery lacks behind other medical and surgical specialties in terms of multi‐investigator collaboration [6]. With the advent of technological advancements accelerated by the COVID‐19 pandemic, virtual collaboration is more accessible than ever and should be leveraged to foster future multi‐centre research efforts [88].

### Citation analysis

Despite increasing interest in the fragility index across orthopaedic surgery, it is imperative to ensure that this body of literature is truly impacting practice. While no significant difference in median citation density was identified across orthopaedic subspecialties, sports medicine presented with the highest metric (median citation density, 4.3; IQR, 1.0–6.6). Moreover, sports medicine articles comprised the greatest proportion of publications both in 2024 (35%), the most recent full year of publications, and overall (25%). This phenomenon is likely multifactorial including both collective interest in the subject matter across sports medicine specialists, as well as external pressures to publish. For example, DeFroda et al. demonstrated that sports medicine fellowship applicants are applying with increasing number of publications [19], with a separate survey study by Baweja et al. noting that publications were the fourth most important factor in assessing an applicant′s candidacy as per sports fellowship directors [3]. Nonetheless, publication volumes must be married with impact, which is evaluated by the h‐index [39]. As demonstrated by Zhu et al., increasing h‐index has been found to be associated with National Institutes of Health (NIH) funding in orthopaedic surgery [113]. Similarly, Chen et al. found that total joint arthroplasty surgeons that secured NIH funding also had a significantly higher mean h‐index compared to those who did not (48.1 vs. 10.4, *p* < 0.001) [12]. Though the h‐index is typically used to assess individual impact, this review performed a similar analysis for this field of study with the h‐index found to be 25, much lower than the calibre of surgeons securing significant governmental research funding. While the h‐index is confounded by the tenure of an academic (i.e., increasing career duration allows for more time to accumulate citations), it is still imperative to critically assess the clinical impact of fragility index research, and whether it is an effective use of resources.

### Limitations

This study is not without its limitations. First, included studies reported the fragility and reverse fragility indices using heterogeneous descriptive statistics. To avoid inaccurate conversions to a single measure of central tendency (e.g., median), we reported the FI and RFI as per index study. This also maximises the number of future studies that may reference their findings against the literature at‐large. Further, various eligibility criteria are used to assess the FI and RFI in orthopaedic surgery with potential overlap in included studies. To minimise this risk, this review stratified studies as either subspeciality‐ or pathology‐specific. Ultimately, this is the largest and most comprehensive study to‐date amalgamating the fragility index literature in orthopaedic surgery, highlighting both key takeaways as well as methodologic concerns moving forward.

## CONCLUSION

The majority of fragility index research is concentrated across a few orthopaedic subspecialties with redundant findings indicating low statistical robustness. Recurring methodologic recommendations based on correlation analyses include increasing patient sample size to increase study power. Methodological recommendations from this body of research should be integrated into future original studies to strengthen statistical robustness.

## AUTHOR CONTRIBUTIONS

Hassaan Abdel Khalik was involved in the study conception, study screening, data abstraction, data analysis, and manuscript writing. Helena Son was involved in the study screening, data abstraction, and manuscript writing. Krishnateja Narayana was involved in study screening and data abstraction. Olufemi Rolland Ayeni and Moin Khan were involved in manuscript writing and review.

## CONFLICT OF INTEREST STATEMENT

Dr. Ayeni reports consulting fees from Stryker Canada, serves as President of the Canadian Orthopaedic Association, is the owner of Notch Academy, and holds a Tier 2 Canada Research Chair. The remaining authors declare no conflict of interest.

## ETHICS STATEMENT

None declared.

## Supporting information

FI in Ortho SR_Supplementary File.

## Data Availability

The data that support the findings of this study are available from the corresponding author upon reasonable request.
