# Supplementary material for: Recurring low statistical robustness in orthopaedic surgery: A systematic review of 84 fragility index studies
Source: J Exp Orthop. 2025 Dec 1;12(4):e70504. doi: 10.1002/jeo2.70504 (PMC12667215; doi:10.1002/jeo2.70504)
Supplement: Supplementary file 1 — FI in Ortho SR_Supplementary File. [file JEO2-12-e70504-s001.docx]

**Supplemental Digital Content Table 1** Search Strategy

| OVID Search Terms for MEDLINE, EMBASE and Cochrane Database of Systematic Reviews |
| --- |
| 1. Fragility index.mp. 2. Fragility quotient.mp. 3. Statistical fragility.mp. 4. Statistically fragile.mp. 5. 1 or 2 or 3 or 4 |

**Supplemental Digital Content Table 2** Summary findings of correlation analyses

|  | Total studies, n (% of all) | p<0.05, n (% of assessing) |
| --- | --- | --- |
| Increasing sample size | 20 (95) | 10 (50) |
| Increasing n of patients lost to follow-up | 12 (57) | 0 (0) |
| Increasing journal impact factor | 10 (48) | 2 (20) |
| Decreasing p-value | 8 (38) | 7 (88) |
| Increasing n of observed events | 5 (24) | 2 (40) |
| Later publication year | 5 (24) | 1 (20) |
| Journal citations | 5 (24) | 1 (20) |
| Risk of bias domains | 3 (14) | 0 (0) |
| Increasing study power | 2 (10) | 1 (50) |
| Article citations | 2 (10) | 0 (0) |
| Relative citation ratio | 2 (10) | 0 (0) |
| Trial funding | 1 (5) | 0 (0) |
